# Association of a longitudinal, preclinical ultrasound curriculum with medical student performance

**DOI:** 10.1186/s12909-022-03108-0

**Published:** 2022-01-21

**Authors:** David A Haidar, Ross Kessler, Neil K Khanna, Michael T Cover, John C Burkhardt, Nik Theyyunni, Ryan V Tucker, Rob D Huang, Elizabeth Holman, Patrick D Bridge, Katherine A Klein, Christopher M Fung

**Affiliations:** 1 Department of Emergency Medicine, University of Michigan, Ann Arbor, Michigan USA; 2 Department of Emergency Medicine, University of Washington, Seattle, Washington USA; 3 Department of Emergency Medicine, Henry Ford Health System, Detroit, Michigan USA; 4 Department of Emergency Medicine, University Hospitals, Cleveland, Ohio USA; 5 Department of Learning Health Sciences, University of Michigan, Ann Arbor, Michigan USA; 6 Office of Evaluation and Assessment, University of Michigan Medical School, Ann Arbor, Michigan USA; 7 Department of Radiology, University of Michigan, Ann Arbor, Michigan USA; 8 Taubman Center, B1-380A 1500 E. Medical Center Dr., SPC 5305, 48109 Ann Arbor, USA MI

**Keywords:** Ultrasound, POCUS, Medical student, Step1, Anatomy, Grades

## Abstract

**Introduction:**

Point-of-care ultrasound (US) is used in clinical practice across many specialties. Ultrasound (US) curricula for medical students are increasingly common. Optimal timing, structure, and effect of ultrasound education during medical school remains poorly understood. This study aims to retrospectively determine the association between participation in a preclinical, longitudinal US curriculum and medical student academic performance.

**Methods:**

All first-year medical students at a medical school in the Midwest region of the United States were offered a voluntary longitudinal US curriculum. Participants were selected by random lottery. The curriculum consisted of five three-hour hands on-sessions with matching asynchronous content covering anatomy and pathologic findings. Content was paired with organ system blocks in the standard first year curriculum at our medical school. Exam scores between the participating and non-participating students were compared to evaluate the objective impact of US education on performance in an existing curriculum. We hypothesized that there would be an association between participation in the curriculum and improved medical student performance. Secondary outcomes included shelf exam scores for the surgery, internal medicine, neurology clerkships and USMLE Step 1. A multivariable linear regression model was used to evaluate the association of US curriculum participation with student performance. Scores were adjusted for age, gender, MCAT percentile, and science or engineering degree.

**Results:**

76 of 178 students applied to participate in the curriculum, of which 51 were accepted. US curriculum students were compared to non-participating students (n = 127) from the same class. The US curriculum students performed better in cardiovascular anatomy (mean score 92.1 vs. 88.7, p = 0.048 after adjustment for multiple comparisons). There were no significant differences in cumulative cardiovascular exam scores, or in anatomy and cumulative exam scores for the gastroenterology and neurology blocks. The effect of US curriculum participation on cardiovascular anatomy scores was estimated to be an improvement of 3.48 points (95% CI 0.78-6.18). No significant differences were observed for USMLE Step 1 or clerkship shelf exams. There were no significant differences in either preclinical, clerkship or Step 1 score for the 25 students who applied and were not accepted and the 102 who did not apply.

**Conclusions:**

Participation in a preclinical longitudinal US curriculum was associated with improved exam performance in cardiovascular anatomy but not examination of other cardiovascular system concepts. Neither anatomy or comprehensive exam scores for neurology and gastrointestinal organ system blocks were improved.

**Supplementary Information:**

The online version contains supplementary material available at 10.1186/s12909-022-03108-0.

## Introduction

### Background

Point-of-care ultrasound (POCUS) is an integral component of patient care and an important aspect of resident education across a variety of specialties such as emergency medicine [1, 2]^,,^ internal medicine [3, 4], family medicine [5], surgery [6], and anesthesiology [7]. POCUS curricula have been developed in medical schools across the country in recognition of the increasingly important role POCUS has across disciplines [8–11]. Undergraduate medical education (UME) POCUS curricula include one-day simulation labs, electives, longitudinal preclinical and clinical courses but these different approaches of POCUS education have had unclear association with medical student performance [11–13].

Students consider POCUS education to be an important part of their clinical education and future practice of medicine [14]. Trainees view POCUS education as valuable and provides them with more confidence in their diagnostic capabilities and ultrasound skills [12, 15, 16]. POCUS education is associated with improved student attitude, confidence, and ability to perform physical exams and improved evaluation of these exam skills in Objective Standardized Clinical Examination (OSCE) scores [17–20]. POCUS education has also been associated with increased student confidence in performance of bedside procedures [19, 21–23]. Kondrashov et al. evaluated the impact of an US course on anatomy knowledge, however a pre- and post-test created specifically for the course was used for assessment [24].

Overall, multiple studies have shown that student comprehension of anatomic concepts improve after completion of an US curriculum but have relied on student survey data as the method of assessment with limited longitudinal evaluation of student performance. A systematic and critical review published in 2017 reported that despite the growing support for POCUS education in UME, there is limited data to objectively express the impact of POCUS education on preclinical assessments and insufficient empirical evidence to substantiate claims of benefit [25]. This has led to calls for further evidence to define the optimal timing and role for ultrasound in UME [26]. Given the limited data, we sought to determine the effects of a longitudinal preclinical ultrasound (US) curriculum on medical student performance.

#### Objectives

Our primary objective was to evaluate the association of a pilot preclinical longitudinal US curriculum with medical student performance in the cardiovascular, gastrointestinal, and neurology preclinical courses; which were divided into the comprehensive and anatomy practical exam scores. Our secondary objectives were to evaluate impact on the internal medicine, neurology and surgery clinical clerkship shelf exams and the United States Medical Licensing Examination (USMLE) Step 1 exam. We hypothesized that participation in the US curriculum would be associated with improved performance in content areas (cardiovascular and gastrointestinal courses) with clear POCUS applications but not in other areas (neurology) where the POCUS curriculum was not as well matched to the course content.

## Methods

### Study Design

This study was determined to be exempt by our institution’s IRB and was approved by the medical school’s Office of Evaluation and Assessment. This study was as a retrospective cohort study conducted at a single medical school in the Midwest region of the United States. The outcomes of interest were medical student performance in preclinical and clinical courses as well as the USMLE Step 1. The primary exposure of interest was participation in an optional preclinical, longitudinal US curriculum that occurred during the students’ first year.

### Study setting and medical school curriculum

Our institution’s medical school has approximately 180 students per class. During this study, the preclinical curriculum was divided into blocks by organ system. The organ systems were divided into cardiovascular, pulmonary, renal, gastrointestinal, hematology/oncology, endocrine/reproduction, musculoskeletal, neurology, dermatology, psychiatry, and infectious diseases blocks. The preclinical curriculum is approximately 1.5 years with students learning normal anatomy, physiology, histology, embryology, pharmacology and pathophysiology within each block. The anatomy lab curriculum ran concurrently within these organ blocks. Students met for in-person cadaver lab sessions one to two times per week. Students began clinical rotations 1.5 years after matriculating to medical school. Core required clinical rotations included internal medicine, surgery, neurology, psychiatry, obstetrics/gynecology, pediatrics, and family medicine. USMLE Step 1 examination occurred at the end of their clinical rotations, which was generally 2.5 years into their training.

### Testing and evaluation

During the preclinical curriculum, students were evaluated with weekly or biweekly online multiple-choice exams followed by an end of block online multiple-choice final exam. The periodic exams included two anatomy questions per exam, and the final exam had two questions per cadaver lab session per block. The final exam was also accompanied by an in-person anatomy practical for which students were required to identify structures in a write-in exam. The anatomy exam score for each block was derived from the in-person practical as the percentage of correct out of total questions. The comprehensive exam score for each block was the percentage correct of all other evaluations including periodic quizzes and final exam, excluding the anatomy practical. During clinical rotations, students are evaluated with standardized National Board of Medical Examiners (NBME) shelf exams with the exception of the neurology exam, which was an institutional exam developed by the neurology clerkship director.

### Selection of participants and predictor variables

**Fig. 1:**
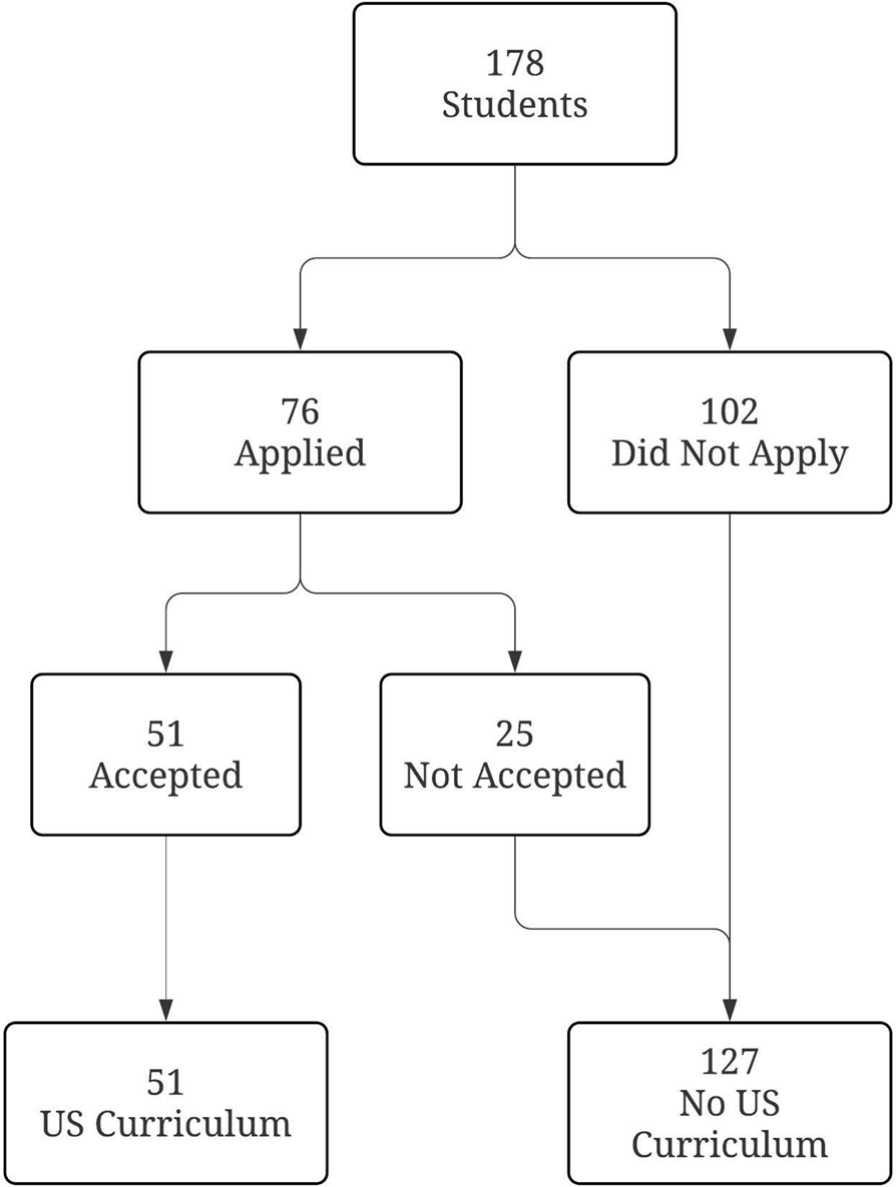
Study flow diagram. Students who applied to the US curriculum were selected at random for 51 available spots. All students matriculated to the same first year medical school class

Shortly after matriculating, all students entering medical school in the fall of 2018 were offered a voluntary longitudinal US curriculum that was paired with their organ system blocks. The optional curriculum was advertised via email to the entire class and two email reminders were also delivered. Students were not asked to specifically decline the curriculum. Students applied for the curriculum and participants were selected by random lottery. Random selection was required because the maximum number of students was limited by physical space and number of instructors. The study flow diagram is detailed in Fig. 1. Students were considered using two different sets of categories. The primary set of comparisons was between students who participated and those that did not. The second set of comparisons, which was conducted as a supplemental analysis, was between three groups: accepted to the US curriculum, applied but not accepted, or did not apply. Acceptance into the program was randomly assigned from all applicants and does not represent selection based on merit. We conducted this second set of comparisons to control for students who might have shown more inherent interest in US and anatomy that might be associated with participation in the US program. For primary analyses age, gender, undergraduate degree, and Medical College Admission Test (MCAT) score percentile were also selected *a priori* as predictor variables.

### Ultrasound curriculum

The selected students participated in five separate three hour hands-on sessions for a total of 15 h of in-person ultrasound education. Attendance by the selected students for all the sessions was mandatory. The five sessions were focused on head & neck, cardiovascular, abdominal, musculoskeletal, and procedural ultrasound. The sessions temporally aligned with the current block in their regular curriculum except for the procedural ultrasound session, which occurred during the students’ neurology block. Topics covered during each session are detailed in Table S1.

Each session was led by ultrasound trained faculty from the Departments of Emergency Medicine (EM) and Radiology with ultrasound trained residents and fellows also serving as small group instructors. Faculty from Radiology were board certified radiologists. Faculty from EM were designated Clinical Ultrasound faculty by the department. Fellows were either board eligible radiologists training in a radiology subspecialty or board eligible emergency physicians in fellowship for advanced emergency ultrasound. Residents leading small groups were senior EM residents participating in the advance ultrasound professional development track. Students performed instructor led practice scans on each other or paid models. Prior to the session, students were sent instructional videos detailing image acquisition and a review of anatomy using ultrasound images. The instructional videos were created by senior medical students with supervision by ultrasound trained faculty and each was less than 10 min in length. During the procedural ultrasound session, they practiced basic bedside procedures such as central and peripheral intravenous line placement on simulators. Students were divided into groups of up to 5 students per instructor for the hands-on practice. For each session, the videos and small groups covered the topics listed in Table S1. Small group instructors provided bedside teaching including guidance on anatomy, external landmarks, machine use, image acquisition technique, and clinical relevance of ultrasound findings.

### Selection of preclinical and clinical block outcomes

Cardiovascular (CV), gastrointestinal (GI) and neurology block outcomes were selected from the preclinical curriculum. CV and GI blocks were matched to ultrasound curriculum hands-on sessions. The neurology block was not matched to an ultrasound session and served as a control. The musculoskeletal block was not included due to limited content overlap with the ultrasound session. We considered preclinical performance to be the primary outcome and within each block we analyzed cumulative block grades and separate anatomy grades. Secondary outcomes included shelf exam scores from the internal medicine, surgery, and neurology clerkships as well as USMLE Step 1 score. These clerkship exams were selected for overlap in content areas with the selected preclinical blocks. Other clinical and preclinical blocks were not selected because they were not matched in content to an ultrasound curriculum session and to limit multiple hypothesis testing.

### Statistical Analysis

Descriptive statistics were performed on all predictor and outcome variables. Continuous variables were described by means with standard deviations and evaluation for statistically significant differences by group using two sample t-tests or one-way analysis of variance for the supplemental three-group comparison. Categorical variables were described by counts with percentages and compared using the chi squared test. Multivariable linear regression was used to determine the association of participation in the US curriculum with preclinical exam scores after adjustment for MCAT percentile, undergraduate science or engineering major, age and gender of the student. Bonferroni correction was used to adjust p-values for six comparisons in the analysis of preclinical exam scores. All statistical analyses were performed in RStudio version 1.2.5 (RStudio, Boston, MA) with R version 3.6.2 (The R Foundation, Vienna, Austria).

## Results

### Student characteristics

One hundred and seventy-eight medical students in the class were studied. Seventy-six (43%) applied for the longitudinal US curriculum and of these, fifty-one (29%) were accepted. These groups are enumerated in the study flow diagram (Fig. 1). There were no statistically significant differences between students who participated in the US curriculum and those that did not for age, gender, science or engineering undergraduate degree or MCAT percentile (Table 1). There were also no significant differences when these characteristics were compared with those students that applied but were not accepted as a separate group (Table S2).

**Table 1:** Student Characteristics

	US Curriculum	No US Curriculum	p-value
Number of students, n (%)	51 (28.7%)	127 (71.3%)	
Age, mean years (SD)	24.9 (2.1)	25 (2.1)	0.696
Female, n (%)	23 (45.1%)	74 (58.3%)	0.153
Science or engineering degree, n (%)	33 (64.7%)	14 (59.1%)	0.597
MCAT percentile, mean (SD)^a^	91 (9.3)	89.5 (10.5)	0.371

^a^3 students in the cohort did not have MCAT scores available

### Preclinical organ block performance

**Fig. 2:**
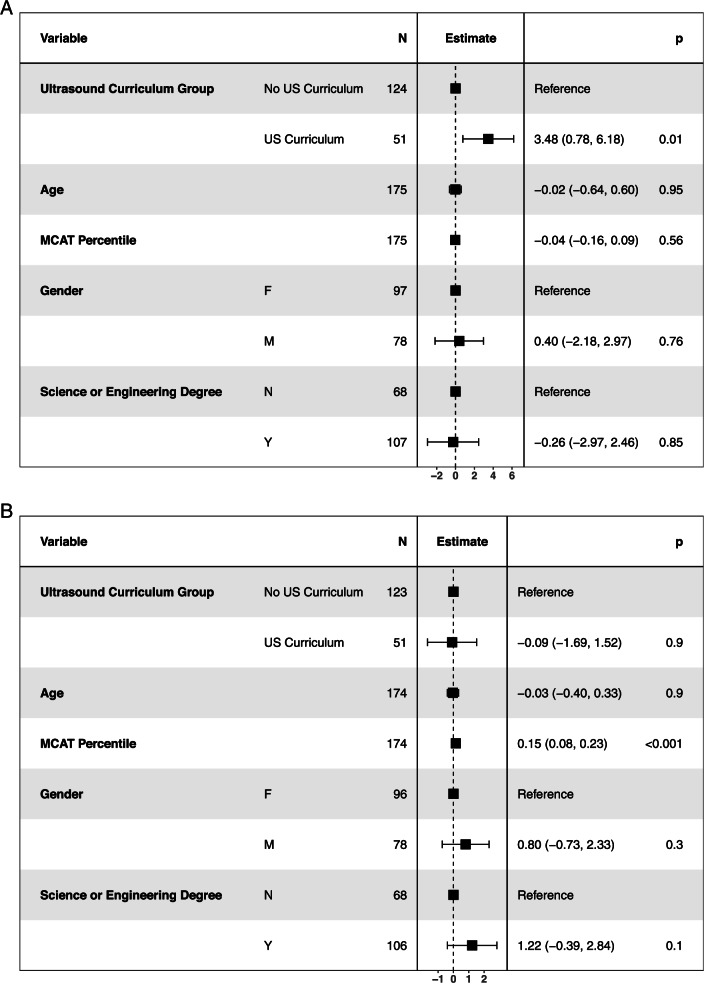
**A** Forest plot for estimate of effect on cardiovascular anatomy score. The estimate effect for each variable is plotted in the middle column. The square represents the point estimate of effect and error bars are the 95% confidence interval. Higher estimate indicates higher score on the cardiovascular anatomy exam. Estimate for age is per year and estimate for MCAT percentile is per percentile point. Three students did not have MCAT scores available and these observations were subject to listwise deletion. MCAT = Medical College Admission Test. **B **Forest plot for estimate of effect on cardiovascular cumulative score. The estimate effect for each variable is plotted in the middle column. The square represents the point estimate of effect and error bars are the 95% confidence interval. Higher estimate indicates higher score on the cardiovascular cumulative exam. Estimate for age is per year and estimate for MCAT percentile is per percentile point. Three students did not have MCAT score available and one student received a zero score on this exam. These observations were subject to listwise deletion. MCAT = Medical College Admission Test

Students who participated in the US curriculum had a higher mean score for the CV anatomy Sect. (92.1 v 88.7, p = 0.008) (Table 2). However, this difference was not seen in the cumulative CV scores. There were no significant differences in either anatomy or cumulative exam scores in the GI and neurology blocks. After correction for multiple comparisons, the difference in mean CV anatomy scores remained statistically significant (p = 0.048). When students that applied but were not accepted were treated as a third separate group, students who participated in the US curriculum continued to have a higher mean score for CV anatomy (Table S3). The association between participation in the US curriculum and CV exam scores was estimated using multivariable linear regression with student age, gender, science or engineering degree status, and MCAT percentile as *a priori* selected covariates (Fig. 2). Participation in the US curriculum resulted in a significant increase in predicted CV anatomy scores (3.48 points, 95% CI 0.78 - 6.18). Other covariates were not associated with a significant effect. For the CV cumulative score, participation did not result in a significant effect although MCAT percentile did predict better performance (0.15 CV cumulative score points per MCAT percentile point, 95% CI 0.08 - 0.23). When students who applied but were not accepted were treated as a separate group in this model (Fig. S1), participation in the US curriculum also predicted higher CV anatomy scores with a similar magnitude (3.18 points, 95% CI 0.38 - 5.98). There was also no difference in CV cumulative scores with unaccepted students as a separate group and MCAT percentile remained predictive of higher CV cumulative score in this model (Fig. S2).

**Table 2:** First year organ block scores

Organ system block, mean score (SD)	US Curriculum	No US Curriculum	Difference in means^c^ (95% CI)	
Cardiovascular - Anatomy	92.1 (7.3)	88.5 (8.3)	3.4 (0.9 - 6.2)^%^	
Cardiovascular - Cumulative^a^	90.5 (4.8)	90.3 (5.3)	0.2 (-1.5 - 1.9)	
Gastrointestinal - Anatomy	84.6 (6.8)	83.5 (8.9)	0.8 (-1.6 - 3.9)	
Gastrointestinal - Cumulative	87.0 (5.1)	86.6 (5.7)	0.2 (-1.4 - 2.2)	
Neurological - Anatomy	81.7 (8.9)	81.5 (8.8)	0.2 (-2.7 - 3.1)	
Neurological – Cumulative^b^	82.8 (5.3)	83.0 (5.1)	-0.2 (-1.9 - 1.5)	

^%^p-value = 0.008 (0.048 after correction for six comparisons)

^a^1 zero score was reported in the Cardiovascular - Cumulative Exam in the No US Curriculum group and excluded

^b^1 zero score was reported in the Neurological - Cumulative Exam in the No US Curriculum group and excluded

^c^No US Curriculum is the reference group for difference in means

### Clinical clerkship shelf exam and USMLE Step 1 performance

Clerkship and USMLE Step 1 scores are detailed Table 3. Students who participated in the US curriculum did not score significantly higher in shelf exams for internal medicine, surgery and neurology. Mean surgery shelf exam scores were higher (77.3 vs. 74.6) for students who participated in the US curriculum although this difference did not reach statistical significance (p = 0.051). Students who participated in the US curriculum also had a mean USMLE Step 1 score that was higher (241.9 vs. 237.3) than students who did not participate in the US curriculum, but this difference was also not statistically significant (p = 0.081).

**Table 3:** Clerkship shelf scores and USMLE Step 1

	US Curriculum	No US Curriculum	p-value
USMLE Step 1, mean score (SD)^a^	241.9 (13.5)	237.2 (17.1)	0.081
Internal medicine shelf exam, mean score (SD)^b^	76.9 (7.7)	75.1 (9.3)	0.360
Neurology shelf exam, mean score (SD)^c^	87.1 (6.0)	86.4 (6.5)	0.212
Surgery shelf exam, mean score (SD)^d^	77.3 (7.0)	74.6 (8.5)	0.051

^a^7 students in the cohort did not have USMLE Step 1 scores available

^b^3 students did not have internal medicine shelf exam scores available

^c^2 students did not have neurology shelf exam scores available

^d^2 students did not have surgery shelf exam scores available

## Discussion

Medical students who participated in the longitudinal US curriculum did not have improved preclinical exam scores in gastrointestinal or neurology preclinical blocks. Within the cardiovascular block, medical students who participated in the US curriculum had improved performance on the anatomy practical. This supports our hypothesis that an US curriculum would be associated with improved performance in blocks where the US application was more closely linked to anatomy; as in the link between echocardiography and cardiac anatomy. Thus, the US curriculum as a supplement to the cardiovascular block may have reinforced the information and improved exam scores. We did not expect the US curriculum to impact neurology scores since there were no US curriculum sessions were dedicated to neurological anatomy. The head and neck session did not cover brain, spine or other neuroanatomy. Contrary to our hypothesis, there were no significant differences in GI anatomy exam scores between US curriculum groups. Given the relatively common application of ultrasound to hepatobiliary disease, we expected improvement in GI anatomy exam scores for students that participated in the US curriculum. However, the GI block content and anatomy exam focused primarily on luminal structures, thus limiting anatomic relevance of common US applications for this block.

There were no statistically significant differences in surgery, internal medicine or neurology shelf exam scores as a result of participation in the US curriculum. On the surgery shelf exam, students who participated in the US curriculum scored better but this difference did not reach statistical significance (77.3 vs. 74.6, p = 0.051). Our US curriculum covered the FAST exam extensively, which provides a practical overview of abdominal anatomy. This correlation could have led to these improved exam scores, as Blackstock et al. previously demonstrated that a dedicated US curriculum led to a better understanding of the focused assessment with sonography in trauma (FAST) [9].

There were also no statistically significant differences in USMLE Step 1 scores associated with participation in the US curriculum (mean 241.9 for those students in the US curriculum vs. 237.3 for students not in the curriculum, p = 0.081). Liu et al. also reported finding no difference associated with participation in the US curriculum for USMLE Step 1 [27]. Contrary to our results, Liu et al. found no difference in anatomy exam scores, but did find an association with improved assessment of physical examination skills [27]. There are several key differences between our study and Liu et al. First, our study examined individual organ block performance rather than anatomy or physiology across multiple organ systems, likely leading to a specific association between the US curriculum and performance. We also evaluated all students as a cohort from a single medical school class rather than small samples from two classes (51 students in the US curriculum in one year vs. 34 total over two years). Indeed, Liu et al. reported heterogeneity in their results across years including anatomy performance which was significantly improved by the US curriculum in one year but not the other. Exams at our institution were considered summative rather than formative which may also have increased individual student motivation to maximize exam performance. Finally, while a common method of assessment, standardized exam scores have been shown to correlate poorly with clinical performance [28] and thus are likely to be limited as a marker of learning success from an US curriculum. Future studies should consider other markers of learning success such as student evaluations in clerkships with significant POCUS use such as emergency medicine, anesthesia and critical care.

### Limitations

Our study was limited in size to a single medical school class entering in the Fall of 2018. Thus, preclinical and clinical performance may have been affected by class specific effects. However, this may also have the effect of limiting heterogeneity from other curriculum and evaluation changes that occur year-to-year. Selection bias may also have impacted our results, as we relied on volunteers to sign up for the ultrasound curriculum. This could lead to a self-selecting population of anatomy-savvy students or higher performing students signing up for this course. However, when comparing students that volunteered for the US curriculum but were not accepted, students that participated in the curriculum continued to outperform both those that did not apply and those that were not selected in CV anatomy (Figure S1). We were not able to provide the US curriculum to all students who applied due to limitations on physical space, equipment and instructors. These resource limitations necessitated random selection of students from the pool that applied.

Our US curriculum included an additional 15 h of structured instruction time but students that did not participate in the US curriculum were not required to attend other structured coursework during this time. This may confound interpretation of our results as some students may have used this additional time for independent study or non-academic activities. Furthermore, students participated in the US curriculum only during their first year and the effect of the US curriculum may wane over subsequent years.

Our study’s generalizability and reliability is subject to some of the same issues as many single-site interventions. While our institution’s medical school uses standardized, best practices based assessments of medical students this is not universal. Specific organ block preclinical exams covering anatomy, physiology and pathophysiology may differ across institutions and by year within institutions. Additionally, our neurology shelf exam is not a nationally standardized exam. Our US curriculum was primarily taught by instructors from the Departments of Emergency Medicine and Radiology leading to a focus on application of ultrasound specific to these two specialties. This may limit the applicability of our results to institutions with similar instructors. Despite these limitations, to our knowledge, this is the first study to evaluate the influence of a longitudinal US curriculum on medical student performance on individual preclinical and clinical courses as well as USMLE Step 1.

## Conclusions

Participation in a preclinical longitudinal US curriculum was associated with improved exam performance in cardiovascular anatomy but not the exam score for a comprehensive exam of other cardiovascular system concepts. Neither anatomy or comprehensive exam scores for neurology and gastrointestinal organ system blocks were improved. Future studies can further evaluate this association with a more expansive curriculum to include renal, musculoskeletal, and obstetric ultrasound and its association with the corresponding preclinical and clinical courses. Additionally, prospective data collection for US curriculum specific effects or use of a control intervention may be required to further elucidate the impact of preclinical US curricula. While further studies across multiple institutions and medical school classes is required, implementation of a dedicated US curriculum early in medical training may improve performance in subsequent preclinical and clinical coursework.

## Supplementary Information

**Additional file 1.**

**Additional file 2.**

**Additional file 3.**

## Data Availability

The data supporting the conclusions of this study are not available to maintain the privacy of student education records.
