# Supplementary figures and images for: Association of a longitudinal, preclinical ultrasound curriculum with medical student performance
Source: BMC Med Educ. 2022 Jan 21;22:50. doi: 10.1186/s12909-022-03108-0 (PMC8780388; doi:10.1186/s12909-022-03108-0)

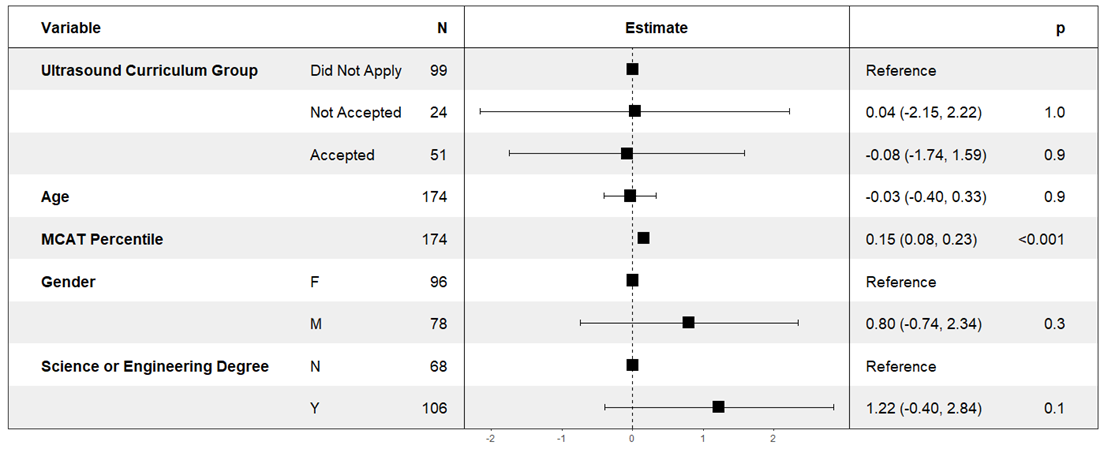

Supplement: Supplementary file 3 — Additional file 3. [file 12909_2022_3108_MOESM3_ESM.png]
